# Prognostic Significance of Psoas Muscle Index in Unresectable Hepatocellular Carcinoma: Comparative Analysis of Lenvatinib and Atezolizumab Plus Bevacizumab

**DOI:** 10.3390/jcm13195925

**Published:** 2024-10-04

**Authors:** Ryuta Shigefuku, Motoh Iwasa, Hideaki Tanaka, Mone Tsukimoto, Yasuyuki Tamai, Naoto Fujiwara, Kyoko Yoshikawa, Masahiko Tameda, Suguru Ogura, Hayato Nakagawa

**Affiliations:** Department of Gastroenterology and Hepatology, Mie University Graduate School of Medicine, 2-174 Edobashi, Tsu 514-8507, Japan; motoh@med.mie-u.ac.jp (M.I.); h.tanaka@med.mie-u.ac.jp (H.T.); mone0428@med.mie-u.ac.jp (M.T.); tamai304051@med.mie-u.ac.jp (Y.T.); naoto-fujiwara@med.mie-u.ac.jp (N.F.); kyoko-y@med.mie-u.ac.jp (K.Y.); tameda@med.mie-u.ac.jp (M.T.); s-ogura@med.mie-u.ac.jp (S.O.)

**Keywords:** hepatocellular carcinoma, atezolizumab plus bevacizumab, lenvatinib, psoas muscle, sarcopenia, survival

## Abstract

**Background and Aims:** Skeletal muscle loss has been identified as a prognostic factor in patients with unresectable hepatocellular carcinoma (uHCC) undergoing treatment with lenvatinib (LEN). While atezolizumab plus bevacizumab (ATZ-BEV) is recommended as first-line therapy for uHCC, the impact of skeletal muscle loss in these patients remains unclear. **Methods:** We enrolled 97 patients treated with either LEN or ATZ-BEV as their first-line therapy and divided them into two groups based on the presence or absence of a low psoas muscle index (low PMI) before treatment. We compared patient characteristics and overall survival (OS) between the groups. Additionally, we investigated the transition of the PMI during drug therapy, specifically before treatment, at the initial evaluation, and after the end of treatment. **Results:** Seventy percent of patients in the LEN group and seventy-one percent in the ATZ-BEV group had a low PMI. Multivariate analysis across all patients revealed a low PMI (hazard ratio [HR] 3.25, *p* = 0.0004) as a prognostic factor for OS. The PMI decreased more in the LEN group compared to the ATZ-BEV group. In the Barcelona Clinic Liver Cancer—C group, the OS of ATZ-BEV therapy was significantly better than that of LEN therapy when a low PMI was present (*p* = 0.046). **Conclusions:** A low PMI emerges as a significant prognostic factor in uHCC patients undergoing drug therapy, not only in LEN therapy but also in ATZ-BEV therapy. Additionally, ATZ-BEV therapy may be more favorable for sarcopenic patients with advanced HCC stages.

## 1. Introduction

Hepatocellular carcinoma (HCC) is a malignancy with a high mortality rate and increasing incidence [1]. Current successful clinical trials of novel anticancer drugs for unresectable HCC (uHCC) have led to the approval of various systemic therapies including multikinase inhibitors of sorafenib (SOR) [2], lenvatinib (LEN) [3], regorafenib [4], and cabozantinib [5] and a monoclonal antibody targeting vascular endothelial growth factor receptor (VEGFR) 2, called ramucirumab [6]. Furthermore, the use of immune checkpoint inhibitors (ICIs) has increased dramatically in the treatment of several types of cancer and has also been confirmed to be an effective treatment for HCC. The IMbrave150 trial [7] and HIMALAYA trial [8] showed that atezolizumab plus bevacizumab (ATZ-BEV) and tremelimumab plus durvalumab (DUR-TRE) achieved notably favorable progression-free and overall survival (OS) compared with SOR in advanced metastatic or uHCC. According to the results of these clinical trials, the first-line drug therapy for advanced HCC has shifted from tyrosine kinase inhibitors, such as SOR and LEN, to ATZ-BEV and DUR-TRE [9].

In HCC, the presence of sarcopenia, which is defined as the progressive and generalized loss of skeletal muscle mass and strength [10], is correlated with poor prognosis [11,12]. In addition, sarcopenia is identified as a prognostic factor for uHCC patients undergoing drug therapy, e.g., SOR [13,14,15] and LEN [16,17,18,19,20,21,22,23,24,25]. However, there is not enough research on the association between sarcopenia and ATZ-BEV therapy. In this study, we investigate the influence of a low psoas muscle index (PMI), which is a phenotype of sarcopenia, on the prognosis of uHCC patients treated with LEN or ATZ-BEV as the first-line drug therapy. Furthermore, we explore the transition of the PMI during treatment, i.e., before, at the initial evaluation, and after the end of treatment.

## 2. Materials and Methods

### 2.1. Regimen of LEN and ATZ-BEV

The dose of LEN was determined by body weight (BW): patients whose BW was <60 kg were given 8 mg per day; and those whose BW was ≥60 kg were given 12 mg per day. Treatment started with a reduced dose that was permitted depending on the patients’ condition. Follow-up visits were scheduled individually at intervals of one to four weeks, depending on the patients’ condition, with blood chemistry being taken. Serum alpha-fetoprotein (AFP) and des gamma-carboxy prothrombin (DCP) were used as liver tumor markers and measured every month. Intravenous ATZ-BEV treatment composed of 1200 mg ATZ plus 15 mg/kg of BW of BEV was administered every 3 weeks.

### 2.2. Assessment of Therapeutic Response to LEN and ATZ-BEV Therapy

The response evaluation criteria in solid tumors (RECIST), ver. 1.1 [26], was used for the evaluation of therapeutic response, which was noted as complete response (CR), partial response (PR), stable disease (SD), progressive disease (PD), objective response rate (ORR; CR + PR), and disease control rate (DCR; CR + PR + SD). The initial assessment of the effect of therapy was performed using dynamic CT results obtained at approximately 6 to 9 weeks after the introduction of LEN or ATZ-BEV whenever possible; then, additional dynamic CT examinations were performed as needed depending on the patients’ condition. After the initial assessment, dynamic CT examinations were performed every 6 to 12 weeks.

### 2.3. Definition of Low Psoas Muscle Index and Timing of Psoas Muscle Index Assessment

The psoas muscle index (PMI) was calculated by dividing the psoas muscle mass at the lumbar vertebral body 3 (cm^2^) by the square of the height (cm^2^/m^2^) using abdominal CT screening, which was performed before the initiation of therapy. The cutoff values of the sarcopenia-related factors were based on the Japan Society of Hepatology guidelines for sarcopenia in liver disease, as this guideline is specifically tailored to patients with liver disease and focuses on the Asian population, defined as a PMI <6.36 cm^2^/m^2^ and <3.92 cm^2^/m^2^ in men and women, respectively [27]. Identification of a low PMI as a prognostic factor let us further explore the changes in the PMI during treatment, including three time points, before treatment (PMI before treatment; 1 to 14 days before treatment), at the initial evaluation (PMI at initial evaluation; six to nine weeks after introduction of drug therapy), and at the end of treatment (PMI at end of treatment; at the last treatment), in the LEN or ATZ-BEV group. In this study, only patients whose PMI could be continuously evaluated by CT scan were included.

### 2.4. Data Collection

The data of the patients’ characteristics were recorded: age, sex, body mass index (BMI) (kg/m^2^), daily alcohol consumption, PMI (cm^2^/m^2^), subcutaneous fat area (SFA) (cm^2^), visceral fat area (VFA) (cm^2^), hepatitis B surface antigen (HBV-Ag), hepatitis C virus antibody (HCV-Ab), total bilirubin (mg/dL), albumin (g/dL), aspartate aminotransferase (AST) (U/L), alanine aminotransferase (ALT) (U/L), prothrombin time (%), platelet count (×10^4^/μL), AFP (ng/mL), DCP (mAU/mL), albumin–bilirubin (ALBI) score [28], ALBI grade [29], Child–Pugh score, Barcelona Clinic Liver Cancer stage (BCLC) [30], macroscopic vascular invasion, and best response (RECIST ver. 1.1). The SFA (cm^2^) and VFA (cm^2^) near the umbilicus were automatically measured by the standard fat attenuation range using the FatScan software program (East Japan Institute of Technology Co., Ltd., Ibaraki, Japan). The subcutaneous fat indices (SFIs) and visceral fat area indices (VFIs) were calculated by dividing the SFA and VFA by the square of the height (cm^2^/m^2^), respectively.

### 2.5. Statistical Analysis

All data are expressed as the median and range. The data were analyzed using the Mann–Whitney U test in two groups. For each continuous variable, the optimal cutoff value that maximized the sum of sensitivity and specificity was selected using the receiver operating characteristic (ROC) analysis for survival. We calculated the cutoff values using the Youden index for ROC analysis. Cumulative OS rates were calculated using the Kaplan–Meier method, and differences between the curves were evaluated using the log-rank test. Survival data were used to establish a univariate and multivariate Cox proportional hazards model. Only variables deemed to be significant (*p* < 0.15) in the univariate analysis were included in the subsequent multivariate analysis. The statistical analyses were performed using the JMP software program (SAS Institute, Cary, NC, USA). Differences were considered significant at *p* < 0.05.

## 3. Results

### 3.1. Patient Characteristics

Forty-one patients treated with LEN and fifty-six patients treated with ATZ-BEV were enrolled in this study, and the characteristics of those who received LEN and ATZ-BEV therapy are shown in Table 1. The ATZ-BEV group had significantly higher DCP levels (*p* = 0.0012) and a higher ORR (*p* = 0.04) compared to the LEN group. A total of 29 out of 41 patients (70.7%) and 40 out of 56 patients (71.4%) were diagnosed with a low PMI in the LEN group and in the ATZ-BEV group, respectively.

Overall, 69 out of 97 patients (71.1%) were categorized into the low-PMI group. This group demonstrated a significantly lower BMI (*p* = 0.0008), a lower SFI (*p* = 0.0016), higher platelet counts (*p* = 0.006), a lower disease control rate (DCR) (*p* = 0.0198), and shorter treatment periods (*p* = 0.0023) compared to the normal-PMI group (Table 2).

A comparison of progression-free survival and overall survival between the LEN group and the ATZ-BEV group was made.

**Figure 1:**
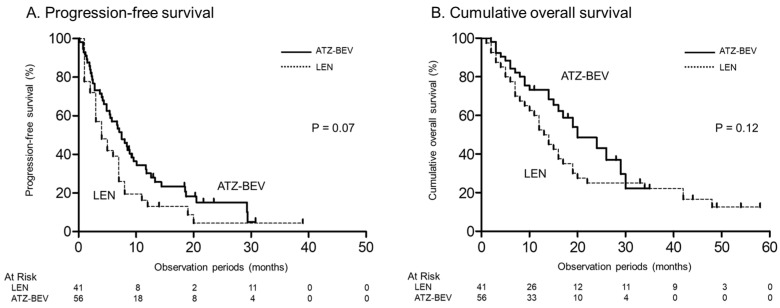
Progression-free survival and cumulative OS curves stratified by treatment group. (**A**) The progression-free survival rates in the LEN therapy (dotted line) and ATZ-BEV therapy (solid line) groups. (**B**) The cumulative OS rates at 6, 12, 18, and 24 months were 77.5%, 52.5%, 35.0%, and 25.0%, respectively, in the LEN therapy group (dotted line), and 84.3%, 73.3%, 58.8%, and 43.2%, respectively, in the ATZ-BEV therapy group (solid line), respectively (*p* = 0.12, log-rank test). OS, overall survival; LEN, lenvatinib; ATZ-BEV, atezolizumab plus bevacizumab.

The median progression-free survival (PFS) for patients treated with LEN and ATZ-BEV was 4.0 and 7.5 months, respectively (*p* = 0.07) (Figure 1A). The median overall survival (OS) times were 13.5 months in the LEN therapy group and 20.0 months in the ATZ-BEV therapy group. The ATZ-BEV group showed a slightly better PFS and overall survival trend, but the difference was not statistically significant (*p* = 0.07, *p* = 0.12) (Figure 1B).

### 3.2. Transition of PMI during Treatment

**Figure 2:**
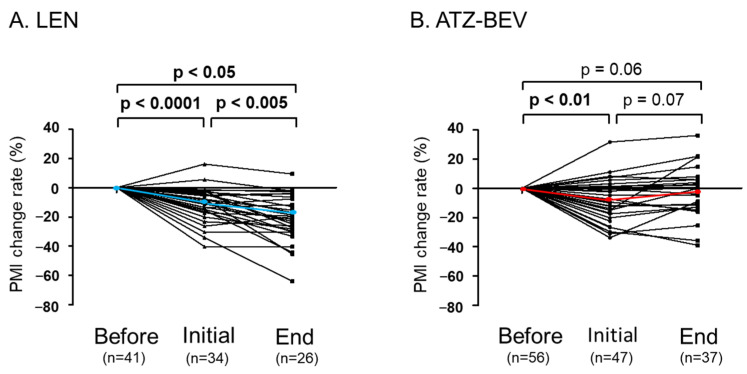
The transition of the PMI before treatment, at the initial evaluation, and at the end of treatment. (**A**) Chronological changes in the PMI in the LEN group. (**B**) Chronological changes in the PMI in the ATZ-BEV group. PMI, psoas muscle index; LEN, lenvatinib; ATZ-BEV, atezolizumab plus bevacizumab; Before, the PMI before treatment; Initial, the PMI at the initial evaluation; End, the PMI at the end of treatment.

The durations of LEN and ATZ-BEV therapies were 7.2 and 7.0 months, respectively (median, *p* = 0.71), indicating no significant difference in the timing of treatment completion between the two therapies. There were significantly more decreased PMI cases in the LEN group (68%) than in the ATZ-BEV group (44%) (*p* < 0.05). In the LEN group, the PMI progressively decreased from the PMI before treatment to the PMI at the initial evaluation and the PMI at the end of treatment (PMI change rate: 0, −12.6%, and −18.0%, respectively) (median: from 4.62 to 4.04 and 3.79 cm²/m², respectively; PMI before treatment vs. PMI at initial evaluation: *p* < 0.0001, PMI at initial evaluation vs. PMI at end of treatment: *p* < 0.05) (Figure 2A). In contrast, in the ATZ-BEV group, the PMI temporarily decreased from the PMI before treatment to the PMI at the initial evaluation (PMI change rate: 0 and −7.4%, respectively) (median: 4.97 and 4.61 cm²/m², respectively; *p* < 0.001), but it recovered by the end of treatment (PMI change rate compared with PMI before treatment: −1.2%) (median: 4.91 cm²/m²) (Figure 2B). These results indicate that the PMI decreased more in the LEN group compared to the ATZ-BEV group.

### 3.3. Cumulative OS According to Having or Not Having Low PMI before Treatment

**Figure 3:**
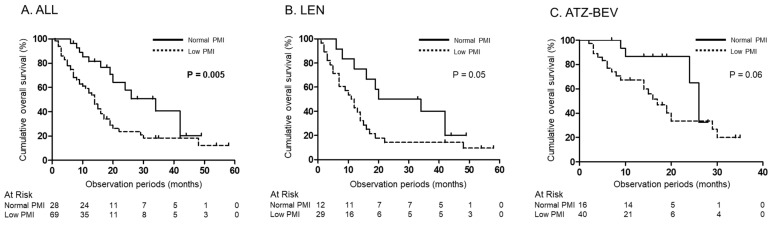
Cumulative OS in uHCC patients based on PMI. (**A**) OS according to having or not having low PMI in all patients. (**B**) OS according to having or not having low PMI in LEN therapy. (**C**) OS according to having or not having low PMI in ATZ-BEV therapy. OS, overall survival; uHCC, unresectable hepatocellular carcinoma; LEN, lenvatinib; ATZ-BEV, atezolizumab plus bevacizumab; PMI, psoas muscle index.

In all patients, the OS of those with a normal PMI was significantly better than that of patients with a low PMI (*p* = 0.005) (Figure 3A). Within the LEN and ATZ-BEV therapy groups, the OS of patients with a normal PMI tended to be better than that of patients with a low PMI (*p* = 0.05, *p* = 0.06), respectively (Figure 3B,C).

**Figure 4:**
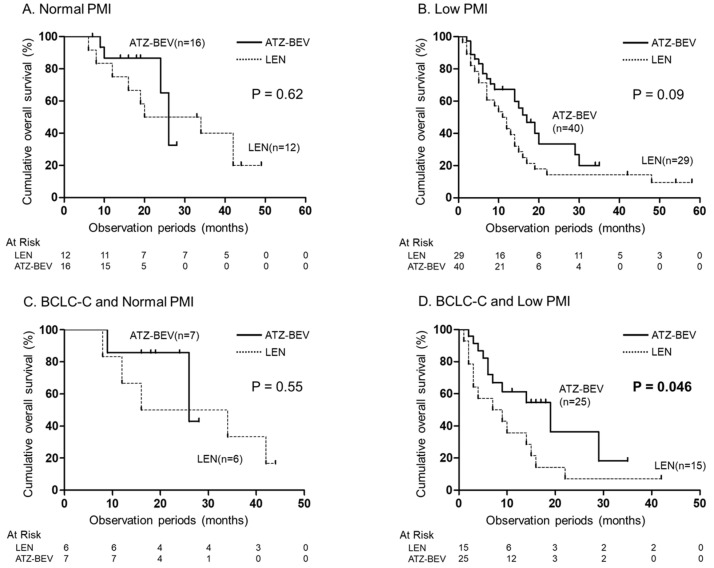
Cumulative OS compared with LEN therapy and ATZ-BEV therapy based on PMI. (**A**) OS in patients with normal PMI. (**B**) OS in patients with low PMI. (**C**) OS in patients with BCLC-C and normal PMI. (**D**) OS in patients with BCLC-C and low PMI. OS, overall survival; LEN, lenvatinib; ATZ-BEV, atezolizumab plus bevacizumab; PMI, psoas muscle index; BCLC-C, Barcelona Clinic Liver Cancer stage C.

A Kaplan–Meier curve comparing OS between LEN therapy and ATZ-BEV therapy based on the presence or absence of a low PMI is shown (Figure 4A–D). In the Barcelona Clinic Liver Cancer (BCLC)—C group, the OS of ATZ-BEV therapy was significantly better than that of LEN therapy when a low PMI was present (*p* = 0.046) (Figure 4D).

### 3.4. Risk Factors Associated with Cumulative OS

In all patients, univariate analysis using the Cox proportional hazards model of the association between OS and patient characteristics showed that the prognostic factors were a low PMI (*p* = 0.002), an ALBI grade 2 or 3 (vs. 1) (*p* = 0.12), and a high DCP level (*p* = 0.04). Multivariate analysis showed that the independent prognostic factor for OS was a low PMI (hazard ratio [HR] 2.57, *p* = 0.0015) (Table 3, part A).

In the LEN group, univariate analysis showed that the prognostic factors were BCLC stage C (vs. B) (*p* = 0.13) and a low PMI (*p* = 0.04). A low PMI was the only independent prognostic factor for OS using multivariate analysis (HR 2.26, *p* = 0.0294) (Table 3, part B). In the ATZ-BEV group, univariate analysis showed that the prognostic factors were a high ALBI grade (*p* = 0.02), a high DCP level (*p* = 0.09), and a low PMI (*p* = 0.02). Using multivariate analysis, the independent prognostic factors for OS were a high ALBI grade (HR 5.17, *p* = 0.0075) and a low PMI (HR 5.02, *p* =0.0020) (Table 3, part C).

In patients with BCLC-C and a low PMI, univariate and multivariate analysis using the Cox proportional hazards model of the association with OS showed that the only favorable prognostic factor was the choice of ATZ-BEV therapy (HR 0.40, *p* = 0.0284) (Table 3, part D).

### 3.5. Relationship between Post-First-Line Therapy and PMI in Transitioning to Subsequent Therapy

The transition rate to subsequent therapy was 21.6% (8 out of 37 cases) in the LEN therapy group and 40.0% (18 out of 45 cases) in the ATZ-BEV group (*p* = 0.18). In the LEN group, four cases (44.4%) with a normal PMI transitioned to subsequent treatment (SOR, ATZ-BEV, cabozantinib, and cisplatin intra-arterial infusion therapy), while four cases (14.3%) with a low PMI transitioned (SOR, ramucirumab, and low-dose 5-FU/cisplatin) (*p* = 0.11). In the ATZ-BEV group, 6 cases (60.0%) with a normal PMI transitioned (5 to LEN and 1 to SOR), while 12 cases (34.3%) with a low PMI transitioned (11 to LEN and 1 to DUR-TRE) (*p* = 0.17).

## 4. Discussion

This retrospective study demonstrated that the presence of a low PMI affects the prognosis of patients treated with not only LEN therapy but also ATZ-BEV therapy. A low PMI has been reported to be a factor related to the poor prognosis in HCC patients treated with LEN [16,17,18,19,20,21,22,23,24,25]. In regard to ATZ-BEV therapy, although it was initially reported that sarcopenia does not determine prognosis [31], current reports have shown that sarcopenia is related to poor progression-free survival [32] and poor prognosis with increased adverse events (AEs) and deterioration of liver function [33]. Moreover, a Japanese multicenter study reported that muscle loss is a factor related to poor prognosis in patients receiving ATZ-BEV [34]. These reports suggest that sarcopenia affects prognosis in ATZ-BEV therapy, and those are consistent with our results.

Our results revealed a progressive decrease in the PMI in the LEN group, whereas the PMI decreased temporarily and recovered after treatment in the ATZ-BEV group. Furthermore, we observed a higher incidence of a decreased PMI in patients treated with LEN compared to those treated with ATZ-BEV during treatment (68% vs. 44%, respectively; *p* < 0.05), indicating that LEN has a significant influence on skeletal muscle. Recent reports have demonstrated that ATZ-BEV therapy induces less skeletal muscle loss compared to LEN therapy, which supports our findings [35,36]. Muscle loss is known to be exacerbated by chemotherapy, including SOR, LEN, and ATZ-BEV. This is consistent with evidence suggesting a role of kinases in the regulation of muscle mass [37,38]. Furthermore, in skeletal muscle stem cells (SkMSCs), fibroblast growth factor receptor 2 (FGFR2) accelerates proliferation and suppresses differentiation through microRNA-271-5p, which directly targets FGFR2 and may regulate the myogenesis of SkMSCs as a myogenesis promoter [39]. The fact that LEN strongly inhibits FGFR2 may have resulted in the reduction in skeletal muscle in our study. Furthermore, AEs such as anorexia (LEN vs. ATZ-BEV: 34 vs. 17.6%), weight loss (31 vs. 11.2%), and fatigue (31 vs. 20.4%) were highly induced in LEN therapy compared to ATZ-BEV therapy, although the overall incidence of AEs was the same in the REFLECT for LEN and IMbrave150 for ATZ-BEV clinical trials 3 7. This higher frequency of AEs in LEN therapy may cause more skeletal muscle loss.

We noticed a tendency toward better OS in patients receiving ATZ-BEV therapy compared to those receiving LEN therapy, particularly in patients with a low PMI (*p* = 0.09). In addition, when limited to patients with a low PMI and BCLC stage C (advanced stage), it was found that LEN therapy had significantly worse prognosis compared to ATZ-BEV therapy. In advanced-stage HCC patients with a low PMI, the choice of ATZ-BEV therapy contributes to the improvement in prognosis. Our results suggest that in sarcopenic patients with an advanced HCC stage, choosing ATZ-BEV treatment, which is less likely to cause a reduction in skeletal muscle mass associated with the treatment, may improve prognosis. This study has a couple of limitations. Firstly, it is a single-center retrospective study with a relatively small study cohort. Secondly, the observation period was not long. Therefore, this study should be validated in larger cohorts of patients at multiple centers over a longer period.

## 5. Conclusions

A low PMI emerged as a significant prognostic factor in both LEN and ATZ-BEV therapies for uHCC. The PMI decreased in both therapies, emphasizing the importance of monitoring body composition during drug therapy for uHCC.

## 6. Patents

This is a retrospective observational study. Prior to the initiation of this study, the study protocol was reviewed and approved by the clinical research ethics review committee of Mie University hospital (H2019-193). Due to a retrospective medical record survey, it was exempt from written or oral consent; however, we released information about this study so patients could opt out of having their data used. This was conducted by reviewing the medical records of 97 patients who were diagnosed with uHCC and treated with LEN between April 2018 and September 2020 and ATZ-BEV between October 2020 and March 2024 as the first-line drug therapy at Mie University Hospital. The HCC diagnoses were based on tumor markers and contrast-enhanced computed tomography (CT) or magnetic resonance imaging of tumors that displayed vascular enhancement in the early phase and washout in the later phase, in accordance with the guidelines of the Japan Society of Hepatology [40].

## Figures and Tables

**Table 1:** Characterization of patients treated with lenvatinib or atezolizumab plus bevacizumab therapy.

	All	LEN	ATZ-BEV	p *
	(*n* = 97)	(*n* = 41)	(*n* = 56)	
Age, year	74.3 (68.9–80.0)	73.0 (64.0–77.5)	75.0 (70.0–81.0)	0.06
Sex, M/W	74/23	29/12	45/11	0.42
BMI, kg/m^2^	23.7 (21.2–25.8)	23.2 (20.1–25.4)	23.5 (21.6–25.9)	0.37
Low PMI: +/− (%)	69/28 (71.1)	29/12 (70.7)	40/16 (71.4)	0.96
PMI, cm^2^/m^2^	4.9 (4.0–6.2)	4.6 (3.7–6.1)	5.0 (4.1–6.3)	0.23
SFI, cm^2^/m^2^	61.4 (39.2–83.3)	59.6 (36.0–80.4)	62.7 (43.1–88.2)	0.41
VFI, cm^2^/m^2^	49.4 (37.5–61.0)	46.4 (36.9–57.7)	52.2 (38.2–61.2)	0.31
Etiology				
HCV/HBV/AL/HCV + AL/ MASLD/others	20/14/25/3/31/4	9/10/10/0/12/0	11/4/15/3/19/4	0.39
Total bilirubin, mg/dL	0.8 (0.6–1.1)	0.9 (0.6–1.2)	0.8 (0.6–1.0)	0.65
Albumin, g/dL	3.6 (3.4–4.0)	3.8 (3.4–4.0)	3.6 (3.4–3.8)	0.24
AST, U/L	46.0 (35.0–61.0)	40.0 (30.0–61.0)	48.0 (35.8–58.8)	0.40
ALT, U/L	28.0 (21.5–41.5)	29.0 (22.0–47.0)	27.5 (20.0–40.0)	0.55
Prothrombin time, %	92.3 (81.4–100.3)	88.0 (75.7–100.0)	92.3 (84.0–99.0)	0.66
Platelet count, ×10^4^/μL	16.5 (11.8–24.7)	16.3 (11.3–24.4)	17.3 (12.0–24.9)	0.37
AFP, ng/mL	30.0 (7.0–1773.6)	18.0 (7.0–1163.0)	48.5 (9.2–1824.7)	0.28
DCP, mAU/mL	542.0 (53.0–8640.0)	142.0 (23.5–1247.0)	1212.0 (86.0–9859.0)	**0.0012**
ALBI score	−2.36 (−2.58–−2.00)	−2.43 (−2.70–−2.11)	−2.31 (−2.52–−1.98)	0.07
Child–Pugh score: 5/6/7/8	52/37/7/1	21/16/3/1	31/21/4/0	0.83
BCLC: B/C	44/53	20/21	24/32	0.60
Vp4: +/− (%)	10/87 (10.3)	2/39 (4.9)	8/48 (14.3)	0.14
Best response (RECIST): CR/PR/SD/PD/ undetermined	6/13/47/22/9	2/2/22/11/4	4/11/25/11/5	-
ORR (CR + PR), *n* (%)	19 (19.6)	4 (12.1)	15 (26.8)	**0.04**
DCR (CR + PR + SD), *n* (%)	66 (68.0)	26 (63.4)	40 (71.2)	0.42
Treatment periods, months	7.0 (3.0–12.1)	7.2 (1.1–13.0)	7.0 (3.0–11.0)	0.71
Observation periods, months	14.0 (6.0–19.0)	13.0 (7.0–22.0)	14.0 (6.0–18.0)	0.40

Note: All data are expressed as the median (first quartile–third quartile). Bold means *p* < 0.05. * Mann–Whitney *U*-test (LEN vs. ATZ-BEV). Abbreviations: LEN, lenvatinib; ATZ-BEV, atezolizumab plus bevacizumab; BMI, body mass index; PMI, psoas muscle index; SFI, subcutaneous fat index; VFI, visceral fat area index; AL, alcohol-associated liver disease; MASLD, metabolic dysfunction-associated steatotic liver disease; AST, aspartate transaminase; ALT, alanine aminotransferase; AFP, alpha-fetoprotein; DCP, des gamma-carboxy prothrombin; ALBI, albumin–bilirubin; BCLC, Barcelona Clinic Liver Cancer; Vp4, main portal vein invasion; RECIST, response evaluation criteria in solid tumors; CR, complete response; PR, partial response; SD, stable disease; PD, progressive disease; ORR, objective response rate; DCR, disease control rate.

**Table 2:** Characteristics of patients with or without low psoas muscle index.

	All	Normal PMI	Low PMI	*p* ^§^
	(*n* = 97)	(*n* = 28)	(*n* = 69)	
Age, year	74.3 (68.9–80.0)	74.3 (67.3–80.5)	74.0 (69.0–79.4)	0.80
Sex, M/W	74/23	17/11	57/12	0.09
BMI, kg/m^2^	23.7 (21.2–25.8)	25.3 (23.2–27.7)	22.6 (20.2–25.2)	**0.0008**
PMI, cm^2^/m^2^	4.9 (4.0–6.2)	6.7 (4.7–7.5)	4.6 (3.5–5.3)	**<0.0001**
SFI, cm^2^/m^2^	61.4 (39.2–83.3)	76.0 (52.2–99.6)	54.8 (35.8–76.4)	**0.0016**
VFI, cm^2^/m^2^	49.4 (37.5–61.0)	51.8 (37.0–61.0)	49.4 (37.7–61.2)	0.75
LEN/ATZ-BEV (*n*) etiology	41/56	12/16	29/40	-
HCV/HBV/AL/HCV + AL/ MASLD/others	20/14/25/3/31/4	7/5/5/0/11/0	13/9/20/3/20/4	-
Total bilirubin, mg/dL	0.8 (0.6–1.1)	0.8 (0.5–1.2)	0.9 (0.6–1.0)	0.46
Albumin, g/dL	3.6 (3.4–4.0)	3.7 (3.3–4.1)	3.6 (3.4–3.9)	0.46
AST, U/L	46.0 (35.0–61.0)	41.5 (31.5–59.0)	47.0 (35.0–62.5)	0.70
ALT, U/L	28.0 (21.5–41.5)	27.5 (19.3–43.5)	29.0 (22.0–41.0)	0.87
Prothrombin time, %	92.3 (81.4–100.3)	94.3 (83.2–105.6)	90.2 (81.0–99.0)	0.37
Platelet count, ×10^4^/μL	16.5 (11.8–24.7)	14.3 (10.6–18.3)	18.9 (12.1–27.9)	**0.0060**
AFP, ng/mL	30.0 (7.0–1773.6)	13.7 (5.3–1865.7)	38.7 (8.0–1539.7)	0.27
DCP, mAU/mL	542.0 (53.0–8640.0)	143.5 (34.8–5855.5)	938.0 (74.0–9324.0)	0.12
ALBI score	−2.36 (−2.58–−2.00)	−2.36 (−2.66–−2.07)	−2.35 (−2.56–−1.97)	0.78
Child–Pugh score: 5/6/7/8	52/37/7/1	16/11/1/0	36/26/6/1	0.62
BCLC: B/C	44/53	15/13	29/40	0.35
Vp4: +/− (%)	10/87 (10.3)	3/25 (10.7)	7/62 (10.1)	0.94
Best response (RECIST1.1): CR/PR/SD/PD/ undetermined	6/13/47/22/9	3/4/18/3/0	3/9/29/19/9	-
ORR (CR + PR), *n* (%)	19 (19.6)	7 (25.0)	12 (17.4)	0.40
DCR (CR + PR + SD), *n* (%)	66 (68.0)	25 (89.3)	41 (59.4)	**0.0198**
Treatment periods, months	7.0 (3.0–12.1)	9.0 (7.1–13.8)	4.0 (2.3–10.8)	**0.0023**
Observation periods, months	14.0 (6.0–19.0)	18.0 (12.5–27.5)	10.0 (4.5–17.0)	**0.0005**

Note: All data are expressed as the median (first quartile–third quartile). Bold means *p* < 0.05. *^§^* Mann–Whitney *U*-test (normal PMI vs. low PMI). Abbreviations: LEN, lenvatinib; ATZ-BEV, atezolizumab plus bevacizumab; BMI, body mass index; PMI, psoas muscle index; AL, alcohol-associated liver disease; MASLD, metabolic dysfunction-associated steatotic liver disease; AST, aspartate transaminase; ALT, alanine aminotransferase; AFP, alpha-fetoprotein; DCP, des gamma-carboxy prothrombin; ALBI, albumin–bilirubin; BCLC, Barcelona Clinic Liver Cancer; Vp4, main portal vein invasion; CR, complete response; PR, partial response; SD, stable disease; PD, progressive disease; ORR, objective response rate; DCR, disease control rate.

**Table 3:** Univariate and multivariate Cox proportional hazards regression analysis for predictors of survival.

**A: All patients with first-line therapy.**						
Predictors	Univariate analysis			Multivariate analysis		
	HR	95%CI	*p* ^†^	HR	95%CI	*p* ^†^
Age (≧75)	0.82	0.4972–1.3572	0.44	0.78	0.4672–1.2848	0.32
Sex (male)	1.07	0.5678–1.8914	0.82	1.66	0.7911–3.3376	0.17
BCLC stage C	1.23	0.7428–2.0517	0.42	1.32	0.7495–2.3384	0.34
High ALBI	1.56	0.8895–2.8642	0.12	1.66	0.9323–3.0960	0.09
High DCP	1.70	1.0232–2.8683	0.04	1.34	0.7711–2.3695	0.30
Low PMI	2.49	1.3865–4.7988	0.002	3.25	1.6542–6.8260	**0.0004**
**B: LEN therapy.**						
Age (≧75)	0.76	0.3842–1.4821	0.42	0.67	0.3288–1.3331	0.25
Sex (male)	1.02	0.5050–2.2348	0.95	2.75	0.9323–8.2031	0.07
BCLC stage C	1.68	0.8542–3.3661	0.13	1.81	0.9153–3.6327	**0.0103**
High ALBI	1.31	0.6512–2.7862	0.46	1.48	0.7096–3.2653	0.30
High DCP	1.61	0.8181–3.2028	0.17	1.01	0.4794–2.0978	0.99
Low PMI	2.13	1.0233–4.8566	0.04	3.38	1.3191–8.3249	**0.0089**
**C: ATZ-BEV therapy.**						
Age (≧75)	1.00	0.4630–2.1831	0.99	0.73	0.3179–1.6643	0.45
Sex (male)	1.11	0.192–2.9615	0.86	1.96	0.4976–6.2238	0.31
BCLC stage C	1.08	0.4952–2.3171	0.85	2.00	0.8523–4.6911	0.11
High ALBI	3.57	1.1945–15.4514	0.02	6.59	1.8150–35.1390	**0.0025**
High DCP	1.96	0.8949–4.5278	0.09	2.35	0.9531–6.2573	0.06
Low PMI	3.01	1.1402–10.3335	0.02	6.83	2.0807–28.9587	**0.0009**
**D: The patients with BCLC-C and a low PMI.**						
Age (≧75)	0.56	0.2493–1.2248	0.15	0.56	0.2331–1.2919	0.17
Sex (male)	2.31	0.5315–7.0435	0.23	3.39	0.7342–11.7979	0.11
High ALBI	1.55	0.6036–4.8585	0.38	1.76	0.6906–5.4192	0.34
High DCP	1.11	0.5029–2.6084	0.80	1.01	0.4257–2.2636	0.61
Selection of ATZ-BEV	0.47	0.2110–1.0101	0.05	0.40	0.1722–0.9076	**0.0284**

^†^ Univariate and multivariate Cox proportional hazards regression analysis. Bold means *p* < 0.05. Abbreviations: LEN, lenvatinib; ATZ-BEV, atezolizumab plus bevacizumab; HR, hazard ratio; 95% CI, 95% confidence interval; BCLC, Barcelona Clinic Liver Cancer; ALBI, albumin–bilirubin; DCP, des gamma-carboxy prothrombin; PMI, psoas muscle index.

## Data Availability

The raw data supporting the conclusions of this article will be made available by the authors on request.

## References

[1] Llovet J.M., Kelley R.K., Villanueva A., Singal A.G., Pikarsky E., Roayaie S., Lencioni R., Koike K., Zucman-Rossi J., Finn R.S. (2021). Hepatocellular carcinoma. Nat. Rev. Dis. Primers.

[2] Llovet J.M., Ricci S., Mazzaferro V., Hilgard P., Gane E., Blanc J.F., de Oliveira A.C., Santoro A., Raoul J.L., Forner A. (2008). Sorafenib in advanced hepatocellular carcinoma. N. Engl. J. Med..

[3] Kudo M., Finn R.S., Qin S., Han K.-H., Ikeda K., Piscaglia F., Baron A., Park J.-W., Han G., Jassem J. (2018). Lenvatinib versus sorafenib in first-line treatment of patients with unresectable hepatocellular carcinoma: A randomised phase 3 non-inferiority trial. Lancet.

[4] Bruix J., Qin S., Merle P., Granito A., Huang Y.-H., Bodoky G., Pracht M., Yokosuka O., Rosmorduc O., Breder V. (2017). Regorafenib for patients with hepatocellular carcinoma who progressed on sorafenib treatment (RESORCE): A randomised, double-blind, placebo-controlled, phase 3 trial. Lancet.

[5] Abou-Alfa G.K., Meyer T., Cheng A.-L., El-Khoueiry A.B., Rimassa L., Ryoo B.-Y., Cicin I., Merle P., Chen Y., Park J.-W. (2018). Cabozantinib in Patients with Advanced and Progressing Hepatocellular Carcinoma. N. Engl. J. Med..

[6] Zhu A.X., Kang Y.-K., Yen C.-J., Finn R.S., Galle P.R., Llovet J.M., Assenat E., Brandi G., Pracht M., Lim H.Y. (2019). Ramucirumab after sorafenib in patients with advanced hepatocellular carcinoma and increased alpha-fetoprotein concentrations (REACH-2): A randomised, double-blind, placebo-controlled, phase 3 trial. Lancet Oncol..

[7] Finn R.S., Qin S., Ikeda M., Galle P.R., Ducreux M., Kim T.-Y., Kudo M., Breder V., Merle P., Kaseb A.O. (2020). Atezolizumab plus Bevacizumab in Unresectable Hepatocellular Carcinoma. N. Engl. J. Med..

[8] de Castria T.B., Khalil D.N., Harding J.J., O’reilly E.M., Abou-Alfa G.K. (2022). Tremelimumab and durvalumab in the treatment of unresectable, advanced hepatocellular carcinoma. Future Oncol..

[9] Kudo M. (2023). Current Therapeutic Strategies for Hepatocellular Carcinoma in Japan. Liver Cancer.

[10] Morley J.E., Baumgartner R.N., Roubenoff R., Mayer J., Nair K.S. (2001). Sarcopenia. J. Lab. Clin. Med..

[11] Fujiwara N., Nakagawa H., Kudo Y., Tateishi R., Taguri M., Watadani T., Nakagomi R., Kondo M., Nakatsuka T., Minami T. (2015). Sarcopenia, intramuscular fat deposition, and visceral adiposity independently predict the outcomes of hepatocellular carcinoma. J. Hepatol..

[12] Tamai Y., Iwasa M., Eguchi A., Shigefuku R., Sugimoto R., Tanaka H., Kobayashi Y., Mizuno S., Nakagawa H. (2022). The prognostic role of controlling nutritional status and skeletal muscle mass in patients with hepatocellular carcinoma after curative treatment. Eur. J. Gastroenterol. Hepatol..

[13] Hiraoka A., Hirooka M., Koizumi Y., Izumoto H., Ueki H., Kaneto M., Kitahata S., Aibiki T., Tomida H., Miyamoto Y. (2017). Muscle volume loss as a prognostic marker in hepatocellular carcinoma patients treated with sorafenib. Hepatol. Res..

[14] Imai K., Takai K., Miwa T., Taguchi D., Hanai T., Suetsugu A., Shiraki M., Shimizu M. (2019). Rapid Depletions of Subcutaneous Fat Mass and Skeletal Muscle Mass Predict Worse Survival in Patients with Hepatocellular Carcinoma Treated with Sorafenib. Cancers.

[15] Yamashima M., Miyaaki H., Honda T., Shibata H., Miuma S., Taura N., Nakao K. (2017). Significance of psoas muscle thickness as an indicator of muscle atrophy in patients with hepatocellular carcinoma treated with sorafenib. Mol. Clin. Oncol..

[16] Fujita M., Takahashi A., Hayashi M., Okai K., Abe K., Ohira H. (2019). Skeletal muscle volume loss during transarterial chemoembolization predicts poor prognosis in patients with hepatocellular carcinoma. Hepatol. Res..

[17] Rinninella E., Cintoni M., Raoul P., Pozzo C., Strippoli A., Ponziani F.R., Pompili M., Bria E., Tortora G., Gasbarrini A. (2020). Skeletal Muscle Loss during Multikinase Inhibitors Therapy: Molecular Pathways, Clinical Implications, and Nutritional Challenges. Nutrients.

[18] Uojima H., Chuma M., Tanaka Y., Hidaka H., Nakazawa T., Iwabuchi S., Kobayashi S., Hattori N., Ogushi K., Morimoto M. (2020). Skeletal Muscle Mass Influences Tolerability and Prognosis in Hepatocellular Carcinoma Patients Treated with Lenvatinib. Liver Cancer.

[19] Hiraoka A., Kumada T., Kariyama K., Tada T., Tani J., Fukunishi S., Atsukawa M., Hirooka M., Tsuji K., Ishikawa T. (2021). Clinical importance of muscle volume in lenvatinib treatment for hepatocellular carcinoma: Analysis adjusted with inverse probability weighting. J. Gastroenterol. Hepatol..

[20] Sugama Y., Miyanishi K., Osuga T., Tanaka S., Hamaguchi K., Ito R., Sakamoto H., Kubo T., Ohnuma H., Murase K. (2021). Combination of psoas muscle mass index and neutrophil/lymphocyte ratio as a prognostic predictor for patients undergoing nonsurgical hepatocellular carcinoma therapy. JGH Open.

[21] Dong D., Shi J.Y., Shang X., Liu B., Xu W.L., Cui G.-Z., Wang N.-Y. (2022). Prognostic significance of sarcopenia in patients with hepatocellular carcinoma treated with lenvatinib: A retrospective analysis. Medicine.

[22] Fujita M., Abe K., Kuroda H., Oikawa T., Ninomiya M., Masamune A., Okumoto K., Katsumi T., Sato W., Iijima K. (2022). Influence of skeletal muscle volume loss during lenvatinib treatment on prognosis in unresectable hepatocellular carcinoma: A multicenter study in Tohoku, Japan. Sci. Rep..

[23] March C., Omari J., Thormann M., Pech M., Wienke A., Surov A. (2022). Prevalence and role of low skeletal muscle mass (LSMM) in hepatocellular carcinoma. A systematic review and meta-analysis. Clin. Nutr. ESPEN.

[24] Yamasaki T., Saeki I., Yamauchi Y., Matsumoto T., Suehiro Y., Kawaoka T., Uchikawa S., Hiramatsu A., Aikata H., Kobayashi K. (2022). Management of Systemic Therapies and Hepatic Arterial Infusion Chemotherapy in Patients with Advanced Hepatocellular Carcinoma Based on Sarcopenia Assessment. Liver Cancer.

[25] Kuo M.-H., Tseng C.-W., Hsu C.-S., Chen Y.-C., Kao I.-T., Wu C.-Y., Shao S.-C. (2023). Prevalence and Effect of Low Skeletal Muscle Mass among Hepatocellular Carcinoma Patients Undergoing Systemic Therapy: A Systematic Review and Meta-Analysis. Cancers.

[26] Eisenhauer E.A., Therasse P., Bogaerts J., Schwartz L.H., Sargent D., Ford R., Dancey J., Arbuck S., Gwyther S., Mooney M. (2009). New response evaluation criteria in solid tumours: Revised RECIST guideline (version 1.1). Eur. J. Cancer.

[27] Nishikawa H., Shiraki M., Hiramatsu A., Moriya K., Hino K., Nishiguchi S. (2016). Japan Society of Hepatology guidelines for sarcopenia in liver disease (1st edition): Recommendation from the working group for creation of sarcopenia assessment criteria. Hepatol. Res..

[28] Johnson P.J., Berhane S., Kagebayashi C., Satomura S., Teng M., Reeves H.L., O’Beirne J., Fox R., Skowronska A., Palmer D. (2015). Assessment of liver function in patients with hepatocellular carcinoma: A new evidence-based approach—The ALBI grade. J. Clin. Oncol..

[29] Kudo M. (2022). Newly Developed Modified ALBI Grade Shows Better Prognostic and Predictive Value for Hepatocellular Carcinoma. Liver Cancer.

[30] Llovet J.M., Villanueva A., Marrero J.A., Schwartz M., Meyer T., Galle P.R., Lencioni R., Greten T.F., Kudo M., Mandrekar S.J. (2021). Trial Design and Endpoints in Hepatocellular Carcinoma: AASLD Consensus Conference. Hepatology.

[31] Toshida K., Itoh S., Tomiyama T., Morinaga A., Kosai Y., Kurihara T., Nagao Y., Morita K., Harada N., Yoshizumi T. (2022). Comparison of the prognostic effect of sarcopenia on atezolizumab plus bevacizumab and lenvatinib therapy in hepatocellular carcinoma patients. JGH Open.

[32] Matsumoto H., Tsuchiya K., Nakanishi H., Hayakawa Y., Yasui Y., Uchihara N., Suzuki K., Tanaka Y., Miyamoto H., Ishido S. (2022). Clinical Usefulness of Monitoring Muscle Volume during Atezolizumab Plus Bevacizumab Therapy in Patients with Unresectable Hepatocellular Carcinoma. Cancers.

[33] Oura K., Morishita A., Manabe T., Takuma K., Nakahara M., Tadokoro T., Fujita K., Mimura S., Tani J., Ono M. (2023). Relationship between Accurate Diagnosis of Sarcopenia and Prognosis in Patients with Hepatocellular Carcinoma Treated with Atezolizumab plus Bevacizumab Combination Therapy. Cancers.

[34] Hiraoka A., Kumada T., Tada T., Hirooka M., Kariyama K., Tani J., Atsukawa M., Takaguchi K., Itobayashi E., Fukunishi S. (2023). Relationship of Atezolizumab plus Bevacizumab Treatment with Muscle Volume Loss in Unresectable Hepatocellular Carcinoma Patients: Multicenter Analysis. Liver Cancer.

[35] Imai K., Takai K., Unome S., Miwa T., Hanai T., Suetsugu A., Shimizu M. (2024). Lenvatinib Exacerbates the Decrease in Skeletal Muscle Mass in Patients with Hepatocellular Carcinoma, Whereas Atezolizumab Plus Bevacizumab Does Not. Cancers.

[36] Chihiro K., Kawaoka T., Uchikawa S., Kinami T., Yamasaki S., Kosaka M., Johira Y., Yano S., Amioka K., Naruto K. (2023). The Analysis of Muscle Volume Measured by Bioelectrical Impedance in Patients with Hepatocellular Carcinoma Treated with First-Line Atezolizumab plus Bevacizumab Combination Therapy or First-Line Lenvatinib. Oncology.

[37] Filbin M.G., Dabral S.K., Pazyra-Murphy M.F., Ramkissoon S., Kung A.L., Pak E., Chung J., Theisen M.A., Sun Y., Franchetti Y. (2013). Coordinate activation of Shh and PI3K signaling in PTEN-deficient glioblastoma: New therapeutic opportunities. Nat. Med..

[38] Antoun S., Birdsell L., Sawyer M.B., Venner P., Escudier B., Baracos V.E. (2010). Association of skeletal muscle wasting with treatment with sorafenib in patients with advanced renal cell carcinoma: Results from a placebo-controlled study. J. Clin. Oncol..

[39] Zhu M., Chen G., Yang Y., Yang J., Qin B., Gu L. (2020). miR-217-5p regulates myogenesis in skeletal muscle stem cells by targeting FGFR2. Mol. Med. Rep..

[40] Kudo M., Kawamura Y., Hasegawa K., Tateishi R., Kariyama K., Shiina S., Toyoda H., Imai Y., Hiraoka A., Ikeda M. (2021). Management of Hepatocellular Carcinoma in Japan: JSH Consensus Statements and Recommendations 2021 Update. Liver Cancer.

